# 2024 Acknowledgment of *Journal of Virology Ad Hoc* Reviewers

**DOI:** 10.1128/jvi.00552-25

**Published:** 2025-05-20

**Authors:** Felicia Goodrum, Stacey Schultz-Cherry

**Affiliations:** 1 University of Arizona College of Medicine 12216 , Tucson, Arizona, USA; 2 Infectious Diseases, St. Jude Children's Research Hospital 5417, Memphis, Tennessee, USA

## EDITORIAL

On behalf of the editors of *Journal of Virology*, we acknowledge the following *ad hoc* reviewers for their time and effort in reviewing manuscripts for us during the past year. Your service is both invaluable and greatly appreciated.

Simona Abbà

Caitlin Abbott

Adly M.M. Abd-Alla

Elsayed M. Abdelwhab

Mohamed Faizal Abdul-Careem

Sahibzada Waheed Abdullah

Chantal Abergel

Jônatas S. Abrahão

Florence Abravanel

Larrea-Sarmiento Adriana

Yongxing Ai

H. A.M Hussein Mohammed AlBayati

Sylvie Alonso

Nihal Altan-Bonnet

Jennifer Altomonte

Diego E. Alvarez

Richard Frederick Ambinder

Maria Joao Amorim

Jing An

Germán Andrés

Erise Anggraini

Simon J. Anthony

Victor Hugo Aquino

Priyamvada Archarya

Armando Arias

Alix Augusto Armero Villanueva

Eurico Arruda

Prabhu S. Arunachalam

Sassan Asgari

Usama Ashraf

Daphne C. Avgousti

Teresa Aydillo

María A. Ayllón

Frank O. Aylward

Faustus Azerigyik

Changming Bai

Camden R. Bair

Christopher C.M. Baker

Steven F. Baker

Siddharth Balachandran

Megan Tierney Baldridge

Anne Balkema-Buschmann

Keir M. Balla

Connor Gavin George Bamford

Hiroshi Bannai

Jingyue Bao

Wenbin Bao

Stefanie Barbirz

Natalia S. Barbosa

John N. Barr

John T. Bates

Lisa Bauer

Fernando Bauermann

Sina Bavari

Victoria K. Baxter

Martin Beer

Antje Beling

Sandrine Belouzard

Jessica Belser

Graham John Belsham

Antonette Bennett

Rudolf K.F. Beran

Eric Bergeron

Stephen Berryman

Frederic Bertels

Antonio Bertoletti

Michael Betts

Darshak Bhatt

David Bhella

Yanmin Bi

Stéphane Biacchesi

Mehdi R. M. Bidokhti

Craig J. Bierle

David M. Bisaro

Paola Blanchette

Jorge Carlos Blanco

Julià Blanco

Boris Bogdanow

Guy Boivin

Paul L. Bollyky

Shelly Bolotin

Diane L. Bolton

Joseph Bondy-Denomy

Mattia Bonsignori

Nitish Boodhoo

Bob Boogaard

Ronen Borenstein

Alexander Borodavka

Jens Bosse

Leticia Botella

Eva Böttcher-Friebertshäuser

Michael J. Bouchard

David Boyd

Shelton S. Bradrick

Susan D. Brain

Jason M. Brenchley

Cecile Breyton

James Brien

Pamela H. Brigleb

Mark A. Brimble

Melinda Ann Brindley

Ditlev E. Brodersen

Lone Brøndsted

Cara E. Brook

Edward P. Browne

Emily A. Bruce

Dunja Bruder

Alejandro Brun

Linda Brunotte

Astrid Bryon

Jens Bukh

Hannah Burgess

Alita R. Burmeister

C. Joaquin Caceres

Sarah Caddy

Ken Cadwell

Tina M. Cairns

Christian Cambillau

Mary Cambou

Rafael K. Campos

Cheng Cao

Jianhao Cao

Yunlong Richard Cao

Gerson Isaac Caraballo

Lars-Anders Carlson

Jeremy Carlton

Mariano Carossino

John P. Carr

Jimena Carrillo-Tripp

Brett Case

Byron Caughey

Shan Cen

Catherine Béatrice Cêtre Sossah

Inho Cha

Supriya Chakraborty

Gary C. Chan

Jasper FW Chan

Martin Chi-Wai Chan

Navaratnarajah Chanakha

Ajit Chande

Kin Chow Chang

Pei-Ching Chang

Theresa L. Chang

Michael S. Chapman

Jeremy Chase Crawford

Jayeshbhai Mansinhbhai Chaudhari

Baoshan Chen

Benjamin Chen

Chun-Hong Chen

Ji-Long Chen

Jiong Chen

Jueqi Chen

Liang Chen

Nanhua Chen

Pei-Jer Chen

Pengfei Chen

Tiansheng Chen

Tzong-Yueh Chen

Weiqiang Chen

Xi Chen

Ying Chen

Zhiwei Chen

Gong Cheng

Xiaoming Cheng

Bor-Luen Chiang

John A. Chiorini

Petr Chlanda

Nam-Hyuk Cho

Kyung (Kay) H. Choi

Young Ki Choi

Rebecca C. Christofferson

Bei-Bei Chu

Hin Chu

Xia Chuai

Javier O. Cifuente

Andrea Cimarelli

Kevin Ciminski

Gino Cingolani

Oya Cingöz

Christiana Victoria Cismaru

Penny Clarke

Matthew H. Collins

Che C. Colpitts

Kristen Lea Conn

Sarah A. Connolly

Kevin M. Coombs

Juliana Reis Cortines

Sara Louise Cosby

Laurent Coscoy

Veronica Paula Costantini

Marceline Cote

Lindsey B. Crawford

Thibaut Crépin

Colin Crump

Sara Cuadrado

Jie Cui

Jun Cui

Junru Cui

Sheng Cui

Anthony L. Cunningham

James Cunningham

Bernadeta Dadonaite

Jinxia Dai

Kai Dallmeier

Robert Daniels

Saumitra Das

Lipsa Dash

Luis LP daSilva

Matt Daugherty

Alan R. Davidson

April Dawn Davis

Patricia M Day

Luana de Borba

Miranda de Graaf

Cornelis A.M. de Haan

Gonzalo de Prat Gay

Charlotte De Rudder

Ricardo de Souza Cardoso

Rik L. de Swart

Erik de Vries

Robert Paul de Vries

Michael Dean

Ward Deboutte

James A. DeCaprio

Nicola Decaro

Jesse D. Deere

Leen Delang

Bernard Delmas

Caroline Demeret

Megan Deming

Liang Deng

Rajat Desikan

Stephanie DeWitte-Orr

Eric Déziel

Arun K. Dhar

Sonali Dhindwal

Francesca di Nunzio

Michael S. Diamond

Adrian Diaz

Isabelle Dietrich

Aurelie Diman

Chan Ding

Richard D. Dix

Thomas Dobner

Terje Dokland

Valerian Dolja

Hu Dong

Wuzi Dong

Xiaofeng Dong

Margherita Doria

Nicole A. Doria-Rose

Nir Drayman

Sarah Dremel

Kristen M Drescher

Marlene Dreux

Ingo Drexler

Jan Felix Drexler

Qian Du

Ruikun Du

Xiangjun Du

Yijun Du

Purnima Dubey

Rebecca M. DuBois

Mariette F. Ducatez

Nisha K. Duggal

Hélène Dutartre

Edward H. Egelman

Catherine Eichwald

Sanford Eigenbrode

Fatma Eldemery

Daniel Elleder

Samantha Emma Elizabeth Ellis

Mark C. Enright

Dean D. Erdman

Lucie Etienne

David H. Evans

David N. Everly

Alex Evilevitch

Katherine J. D. Excoffon

Wenchun Fan

Liurong Fang

Ying Fang

Mazigh Fares

Tibor Farkas

Ten Feizi

Jens Fels

Li Feng

Wen-hai Feng

Gianmarco Ferrara

Matthew J. Ferrari

Claudia V. Filomatori

Stefan Finke

Nicole Fischer

Ian Fish

Louis Flamand

Michelle Flenniken

Ervin Fodor

Suan-Sin Foo

Adriana Forero

Diego Forni

Amelie Fradet-Turcotte

Joe Francica

Oliver I. Fregoso

Graham Lee Freimanis

Juliana Freitas-Astúa

Eva Friebertshaeuser

Jelke Jan Fros

Yuguang Fu

Yu-Zhi Fu

Walter Fuchs

Katsuhiko Fukai

Hideto Fukushi

Fred J. Fuller

Morgan Gaia

Emily N. Gallichotte

Jean-Luc Gallois

Andrea Gamarnik

Don Brad Gammon

Ketaki Ganti

Soren Gantt

Fei Gao

Feng Gao

Yulong Gao

Antonio Garmendia

Robert F. Garry Jr.

Matthew Gartner

Peter J. Gaskill

Yves Gaudin

Eleanor Gaunt

Adam Gehring

Sylvie German-Retana

Benjamin Gewurtz

Jason J. Gill

Sebastian Gisder

Britt A. Glaunsinger

Marie Glenet

Sophie Gobeil

Paul A. Goepfert

Christopher M. Goins

Nandan S. Gokhale

Daniel H. Goldhill

Eduardo Gomez-Casado

Peng Gong

Jose L. Gonzales

Irene González-Domínguez

Daniel Gonzalez-Dunia

James L. Goodson

Karine Gousset

Reingard Grabherr

Laura Graf

Sheila V. Graham

Lisa Gralinski

Todd J. Green

Jonathan S. Griffiths

Alba Grifoni

Martin H. Groschup

Julianne H. Grose

Allison Groseth

Wuxiang Guan

Severin Gudima

Susana Guix

Bronwyn Gunn

Huldrych F. Gunthard

Changjun Guo

Chunhe Guo

Guijie Guo

Rui Guo

Yu Guo

Bart L. Haagmans

Susan Hafenstein

Bumsuk Hahm

Alexander Siegfried Hahn

Rong Hai

Hillel Haim

Reza Hajimorad

Peter Halfmann

Mohammad Halim

Roy A. Hall

Stephan Halle

Barbara Han

Jun Han

Timm C. Harder

Whitney E. Harrington

Audray K. Harris

Robert L. Harrison

Amy L. Hartman

Roland Karl Hartmann

Theodora Hatziioannou

Vasili Hauryliuk

Biao He

Bin He

Feng He

Hongbin He

Qigai He

Nagendra R. Hegde

Marco Hein

Manfred Heinlein

Jan Hellert

Emily Hemann

Andrew Henderson

David Heppner

Jesus Hernandez

Pedro Hernandez

Luis Hernández-Pelegrín

Bobby Brooke Herrera

Roland Herzog

J. Bernard Heymann

Yuta Hikichi

David Hildeman

Rolf Hilgenfeld

Catarina E. Hioe

Alec J. Hirsch

Ryan Hisner

Donata Hoffmann

Melle Holwerda

Angelique Hölzemer

Mei Hong

Jay Hooper

Matthias Horn

Hak Hotta

Chunsheng Hou

Gaopeng Hou

Charlotte Houldcroft

Eike Hrincius

Wei-Li Hsu

Hui Hu

Wenhui Hu

Yunzhang Hu

Zhihong Hu

Zhiqiang Hu

Jinhai Huang

Xiaohong Huang

Xuewei Huang

Yao-Wei Huang

Yong Huang

Natalia Hubbs

Amy W. Hudson

Adam Joseph Hume

Edward Hutchinson

Seungmin Hwang

Jennifer Lynn Hyde

Alexander P. Hynes

Kiwamu Hyodo

Joseph M. Hyser

Thomas Iftner

Terumasa Ikeda

Tetsuro Ikegami

Jean-Luc Imler

Michael J. Imperiale

Darrell J. Irvine

Stuart Isaacs

Masanori Isogawa

Hirotaka Ito

Tijana Ivanovic

Luke R. Iwanowicz

Akiko Iwasaki

Makio Iwashima

Robert Jackson

Terry Jackson

William T. Jackson

Timothy James

Eric Jan

Rohit K. Jangra

Andrew B. Janowski

Paul Jardine

Keith William Jarosinski

Hye Won Jeong

Dawei Jiang

Ping Jiang

Yousheng Jiang

Zhongyi Jiang

Meilin Jin

Eric C. Johannsen

Cecilia Johansson

Clare Jolly

Jennifer Jones

Colleen B. Jonsson

Joyce Jose

Nolwenn Jouvenet

Chen Jian Jun

Kwonil Jung

Sandra Junglen

Li Junping

Nicole Kallewaard

Wael Kamel

Soowon Kang

John Karijolich

Sudhir Pai Kasturi

Hiroshi Katoh

Konstantina Katsarou

Leah C. Katzelnick

Barbara Katzenback

Benedikt Kaufer

Keigo Kawashima

Satoshi Kawato

Mary F. Kearney

Alyson A. Kelvin

Scott P. Kenney

Jeremy R. Keown

Atif Khan

Rajiv Khanna

Reza Khayat

Frederick S. B. Kibenge

Marjolein Kikkert

Baek Kim

Dokyun Kim

Janine Kimpel

Ericka Kirkpatrick Roubidoux

Alex Kleinpeter

Kim D. Klonowski

Thomas Klose

Niklas Klusch

Oren Kobiler

Gary P. Kobinger

Abimbola O. Kolawole

Denis Kolbasov

Anastasia Komarova

Renate König

Eugene V. Koonin

Jennifer Houle Kopanke

Anna Koslova

Dmitry Kostyushev

Sergei V. Kotenko

Verena Krähling

Veronika Krchlíková

Sebastian Kreimendahl

Dimitry N. Krementsov

Mart Krupovic

Ersheng Kuang

Meta Kuehn

Anil Kumar

Deepak Kumar

Rei-Lin Kuo

Yen-Wen Kuo

Sascha Young Kupke

Constantinos Kyriakis

Jason T. Ladner

Michael Lagunoff

Chih-Jen Lai

Benjamin Lamp

Ulrike Lange

Stephanie N. Langel

Agnieszka Łątka

Kathlyn Laval

Mansun Law

Cyril Le Nouën

Valerie Le Sage

Chung-Young Lee

Ji-Seon Lee

Qihan Lee

Wen Shi Lee

Jian Lei

Julian L. Leibowitz

Petr G. Leiman

Scott Leisner

Niels A. W. Lemmermann

Nicholas J. Lennemann

Deborah J. Lenschow

Jiann-Horng Leu

Sebastien Lhomme

Bin Li

Chao Li

Chengjun Li

Dawei Li

Fangfang Li

Guohui Li

Jianrong Li

Jida Li

Kui Li

Ningqiu Li

Pengfei Li

Su Li

Tingdong Li

Wenhui Li

Yize Henry Li

Yuxing Li

Guoxin Liang

Huanhuan Liang

Jiankai Liang

Yuying Liang

Sebastian Liebe

Gaetan Ligat

Cheng-Wen Lin

Roger Lippé

Bing Liu

Guangliang Liu

Helene Minyi Liu

Jianyun Liu

Jun Liu

Lu Liu

Pinghuang Liu

Shengwang Liu

Wanhong Liu

Xing Liu

Namal P. Liyanage

Jason S. Long

Carolina B. Lopez

Jesus Lopez

Tomás López

Niels Lorenzen

Alessio Lorusso

Jonathan Lovell

Rosa Lozano-Duran

Hongzhou Lu

Zhengchun Lu

Jasmina M. Luczo

David M. Lukac

Valeria Lulla

Dahai Luo

Ming Luo

Shuhong Luo

Sabrina Lusvarghi

Huibin Lv

Shawn Lyons

Spyros Lytras

Feng Ma

Wentao Ma

Zhe Ma

Robin M. MacDiarmid

Daniel Macedo de Melo Jorge

Dixie Mager

Sonalika Mahajan

Andrea Maisner

Simon A. Mallal

Rama K. Mallampalli

Daniel Malouli

Carlos Maluquer de Motes

Joao Filipe Inacio Mamede

Shahid Mansoor

Sari Mäntynen

Mark Manzano

Qunying Mao

Kevin Maringer

Verónica Martín

Miguel A. Martín-Acebes

Luis Martinez-Gil

Encarnación Martínez-Salas

Mathias Martins

Mauricio A. Martins

Laura Martin-Sancho

Shin-Yi Lee Marzano

Tetsuro Matano

Mathieu Mateo

Enric M. Mateu

Mauricio G. Mateu

Emilyn E. Matsumura

Keita Matsuno

Eiko Matsuo

Ayumi Matsuyama

Karen L. Maxwell

Jared May

Uri Mbonye

Kevin McCarthy

Christopher John McCormick

John T. McCrone

Michael McFadden

Dorian McIlroy

Gerald Michael McInerney

Jane A. McKeating

Áine McKnight

Paul J. McLaren

Jason S. McLellan

Ryan P. McNamara

Michael A. McVoy

Susanne Meile

Vineet D. Menachery

Jason Mercer

Andres Merits

Andreas Meyerhans

David K. Meyerholz

Eleftherios Michailidis

Pascal Miesen

William E. Miller

Wongi Min

Jennifer F. Moffat

Peter Moffett

Kareem N. Mohni

Ian J. Molineux

Illich Manfred Mombo

Louise Moncla

Nathaniel J. Moorman

Marc Morais

Iain M. Morgan

Yasuko Mori

Kazuhiro Morishita

Eiji Morita

Hiromitsu Moriyama

Koki Morizono

Joseph Mudd

Frauke Muecksch

Elke Mühlberger

Lubbertus C.F. Mulder

Janis A. Müller

Christian Munz

Makoto Murakami

Takayuki Murata

Pablo R. Murcia

Benoît Muylkens

Jillian Myers

Lieve M.J. Naesens

Hiroyuki Nakai

Aarthi Narayanan

Sonia Navas-Martin

Jessica Neil

Martha I. Nelson

Uri Neri

Christopher Lewis Netherton

Christopher Neufeldt

Benjamin W. Neuman

Michael M. Nevels

Timothy Newsome

Songwei Ni

Zhangyong Ning

Xiaoyu Niu

Zheng Niu

David Onalenna Nkwe

Aitor Nogales

Christopher C. Norbury

Shahideh Nouri

Richard Novak

Jurg P. Nuesch

Mylene Ogliastro

Molly Ohainle

Taro Ohkawa

Hiroaki Okamoto

Gene G. Olinger

Alejandro Olmedo-Velarde

Yaw Shin Ooi

Antonius G.P. Oomens

Robert C Orchard

Lucia Ortiz

Fernando A Osorio

Mario Ostrowski

Megha Padi

Cara T. Pager

Jean-Christophe Paillart

Mark Painter

Laura A. Palomares

Søren R. Paludan

Dongli Pan

Jianjun Pan

Tao Pan

Xiaoyi Pan

Marie Pancera

Amanda R. Panfil

Dominic Paquin-Proulx

Mark S. Parcells

Tae Kwann Park

Gabriel I. Parra

Matthew S. Parsons

David Pasdeloup

Matthew D. Pauly

Angela Pearson

Malik Peiris

Philip Pellet

Guiqing Peng

Xiaozhong Peng

Judit Penzes

Alan S. Perelson

Rushika Perera

Mariano Pérez-Filgueira

Patricia A. Pesavento

Marine J. Petit

Stephanie Pfaender

Christian Karl Pfaller

Stacia Phillips

Matthew Pickin

Pedro A. Piedra

Theodore C. Pierson

Madalena Pimentel

Pablo Piñeyro

Daniel D. Pinschewer

Amelia Kahler Pinto

Massimo Pizzato

Pavel Plevka

Stefan Poehlmann

Joe Pogliano

Marie O. Pohl

Leo LM Poon

Welkin H. Pope

Matteo Porotto

Robert David Possee

Jan Postberg

Ultan F. Power

Ann M. Powers

Vidya Mangala Prasad

Lakshmanane Premkumar

Alexander M. Price

David Price

David Prikryl

Juan Pu

Nicole E. Putnam

Xiaole Qi

Xuefeng Qi

Ping Qian

Cheng-Feng Qin

Enya Qing

Xusheng Qiu

Yang Qiu

Diego Quito-Avila

Masmudur M. Rahman

Jaya Rajaiya

Ricardo Rajsbaum

Holly Ramage

Marie-Anne Rameix-Welti

Mangala Rao

Venigalla Rao

Angela L. Rasmussen

Anusyah Rathakrishnan

Sanjay M. Reddy

Alec James Redwood

Matthew Reeves

Keith Reeves

Yujie Ren

Peter Reuther

Felix A. Rey

Stephen A. Rice

Alexsia Richards

Juergen A. Richt

Guus F. Rimmelzwaan

Carmen Rivas

Jacques Robert

David Robertson

Erle Robertson

Barry Rockx

Eduardo Rodríguez-Román

José M. Rojas

Nicolas Romero

Lijun Rong

Amy Rosenfeld

Maria Rosenthal

Ted M. Ross

Cyprian C. Rossetto

Philippe Roumagnac

Ignacio Rubio-Somoza

Alessia Ruggieri

Nicolas Ruggli

Robertus W. Ruigrok

Alistair Russell

Eugene V. Ryabov

Randy E. Sacco

Catherine Sadzot-Delvaux

Margarita Sáiz

Yoshihiro Sakoda

Maria-Carla Saleh

Irene Salinas

Bhargava Teja Sallapalli

Suryaprakash Sambhara

Clare E. Sample

Anna Sanawati

Yongming Sang

Martin Sapp

Bevan Sawatsky

Georgia Schafer

David Scheibner

Mario Schelhaas

Joshua T. Schiffer

Mark R. Schleiss

Anthony P. Schmitt

Mirco Schmolke

Marion Schoof

Michael Schotsaert

Jason Schrad

Gideon Schreiber

Sabrina Schreiner

Thomas F. Schulz

Olivier Schwartz

Stefan Schwartz

Roland Schwarzer

Yogesh Scindia

Kimberley D. Seed

Essen Sefik

Jin-Young Seo

Emily Serman

Ruth Serra-Moreno

Yingli Shang

Jian-zhong Shao

Ling Shao

Deepa K. Sharma

Namisha Sharma

Neelam Sharma-Walia

Hongxing Shen

Wei Shen

Nathan M. Sherer

Lee Sherry

Mang Shi

Eui-Cheol Shin

Hyunjin Shin

Woo-Jin Shin

Makino Shinji

Mai Shiokawa

Hiroaki Shirafuji

Ikuo Shoji

Deepak Shukla

Rafat Siddiqui

Christian Sieben

Thomas J. Silhavy

Peter Simmonds

Amy C. Sims

Pawan Kumar Singh

Rebecca L. Skalsky

Michael A. Skinner

Richard Sloan

Everett Clinton Smith

Thomas E. Smithgall

Sigrun Smla

Ray T.Y. So

Ashley Sobel-Leonard

Dorothy Sojka

Samantha Standish Soldan

Hao Song

Syriam Sooksawasdi Na Ayudhya

Stanislav V. Sosnovtsev

Sandra Souto Pereira

William M. de Souza

Konstantin Maria Johannes Sparrer

Alexandra Spencer

Emily Speranza

Chelsey C. Spriggs

Rajagopalbabu Srinivasan

Richard James Stanton

Tyler Starr

Shaun Stauffer

Spyridon Stavrou

Eike Steinmann

Peter Stern

Noam Stern-Ginossar

Christopher C. Stobart

Peter Stockly

Michael Ray Strand

Blair L. Strang

Patrick Michael Stuart

Jianguo Su

Sundharraman Subramanian

Dongbo Sun

Honglei Sun

Jinfu Sun

Rong Sun

Weina Sun

Xiangjie Sun

Xiulian Sun

Yan Sun

Tetsuro Suzuki

Trevor Sweeney

Katalin Szászi

Steven M. Szczepanek

Takanobu Tagawa

Andrew W. Tai

Kazuo Takayama

Makoto Takeda

Shigeki Takeda

Tomokazu Tamura

Chee Wah Tan

Hua Tang

Liudi Tang

Qiyi Tang

Yan-Dong Tang

Pan Tao

Yizhi J. Tao

Pradip Tarafdar

Stefan Taube

Norbert Tautz

Geraldine Taylor

John Teijaro

Jose G. Teodoro

James M. Termini

Andrea K. Thoma-Kress

Stephen J. Thomas

Lucy G. Thorne

Ji Tianxing

Chih-Feng Tien

Natasha Louise Tilston-Lunel

John C. Tilton

Domenico Tortorella

Nadia Touil

Martha Triantafilou

Jakob Trimpert

Shashank Tripathi

Uwe Truyen

Long Ping Victor Tse

Athe M.N. Tsibris

Jiagang Tu

Massimo Turina

Mudit Tyagi

Kenneth L. Tyler

Nathan A. Ungerleider

Chitra Upadhyay

Jason W. Upton

Andrew Vaillant

Sophie Valkenburg

Carolien van de Sandt

Lia van der Hoek

Paul van der Schoot

James L. Van Etten

Carine Van Lint

Linda van Oosten

Mark J. van Raaij

Pol Scott B. Vande

Elst Niels Vander

Thiru Vanniasinkam

Arvind Varsani

Michael Veit

Hana Veler

Meghan S. Vermillion

Gijs Versteeg

Elisa Vicenzi

Georgios Vidalakis

Abel Viejo-Borbolla

Marco Vignuzzi

Maija Vihinen-Ranta

Jan Vinje

Romain Volmer

Asisa Volz

Jens von Einem

Max von Kleist

Sudhanshu Vrati

Hiep L. X. Vu

Adam Waickman

Kousho Wakae

Xiu-Feng Wan

Bin Wang

Che-Yen Joseph Wang

Hao-Ching Wang

Hui Wang

Jian-Hua Wang

Jie Wang

Jin-Xing Wang

Jun Wang

Kam-Bo Wang

Leyi Wang

Manli Wang

Qiuhong Wang

Shaowen Wang

Tian Wang

Wei-Kung Wang

Xi Wang

Xian-Bing Wang

Xian-Wei Wang

Xiao-Wei Wang

Xiuqing Wang

Yiping Wang

Brian M. Ward

Brian R. Wasik

Koichi Watashi

Michael Way

Friedemann Weber

Wei Wei

Sebastian Weigang

John Weiland

Jason B. Weinberg

Daniela Weiskopf

Carol D. Weiss

Stephen Robert Welch

Kuo-Feng Weng

Jillian N. Whelan

Simon White

Steve Whitham

Matthew S. Wiebe

Kevin Wiehe

Thomas E. Wileman

Harry Williams

Mark R. Wills

Harald Wodrich

Sook-San Wong

Charles Wood

Antoni Wrobel

Jianjun Wu

Weimin Wu

Zhiwei Wu

Brand Meghan Wymore

Ning-Shao Xia

Yuchen Xia

Ye Xiang

Chuan Xiao

Shaobo Xiao

Shuqi Xiao

Yihong Xiao

Xuping Xie

Zhengde Xie

Tian Tian Xu

Wei Xu

Xingang Xu

Fei Yan

Ming Yan

Jian Yang

Kai Yang

Qian Yang

Wan-Shan Yang

Jian Ye

Jing Ye

Hui-Ling Yen

Soner Yildiz

John Yin

Sunnie Yoh

Dongwan Yoo

Kyoung-Jin Yoon

Ian A. York

Xiangbin You

Ry Young

Jacob Yount

Hai Yu

Jingyou Yu

Tao Yu

Xu G. Yu

Xue-jie Yu

Junfa Yuan

Allan Zajac

Jose Luis Zambrano

Marija Zaric

Veronika I. Zarnitsyna

Francisco Murilo Zerbini

Junjie Zhang

Leiliang Zhang

Luwen Zhang

Wei Zhang

Wenyan Zhang

Xiaobo Zhang

Yong-An Zhang

Yong-Zhen Zhang

Zecai Zhang

Dongming Zhao

Jun Zhao

Qin Zhao

Aihua Zheng

Haixue Zheng

Hui Zheng

Jian Zheng

Jie Zhong

Bin Zhou

Jiyong Zhou

Tongqing Zhou

Xiaoling Zhou

Yan Zhou

Yu Zhou

Fei Zhu

Jian Zhu

Ping Zhu

John Ziebuhr

Joseph M. Ziegelbauer

Gert Zimmer

Adam Zlotnick

Seth Zost

Fedor Zurabov

Mark P. Zwart

